# Immunometabolic alterations in type 2 diabetes mellitus revealed by single-cell RNA sequencing: insights into subtypes and therapeutic targets

**DOI:** 10.3389/fimmu.2024.1537909

**Published:** 2025-01-14

**Authors:** Huahua Li, Lingling Zou, Zhaowei Long, Junkun Zhan

**Affiliations:** ^1^ Department of Geriatric, Hunan Provincial People’s Hospital, The First Affiliated Hospital of Hunan Normal University, Changsha, China; ^2^ Department of Geriatric, The Third Affiliated Hospital of Kunming Medical University, Kunming, China; ^3^ Department of Geriatrics, The Second Xiangya Hospital, Central South University, Changsha, China; ^4^ Institute of Aging and Age-related Disease Research, Central South University, Changsha, China

**Keywords:** type 2 diabetes mellitus (T2DM), single-cell RNA sequencing, immunometabolism, T cells, machine learning models

## Abstract

**Background:**

Type 2 Diabetes Mellitus (T2DM) represents a major global health challenge, marked by chronic hyperglycemia, insulin resistance, and immune system dysfunction. Immune cells, including T cells and monocytes, play a pivotal role in driving systemic inflammation in T2DM; however, the underlying single-cell mechanisms remain inadequately defined.

**Methods:**

Single-cell RNA sequencing of peripheral blood mononuclear cells (PBMCs) from 37 patients with T2DM and 11 healthy controls (HC) was conducted. Immune cell types were identified through clustering analysis, followed by differential expression and pathway analysis. Metabolic heterogeneity within T cell subpopulations was evaluated using Gene Set Variation Analysis (GSVA). Machine learning models were constructed to classify T2DM subtypes based on metabolic signatures, and T-cell-monocyte interactions were explored to assess immune crosstalk. Transcription factor (TF) activity was analyzed, and drug enrichment analysis was performed to identify potential therapeutic targets.

**Results:**

In patients with T2DM, a marked increase in monocytes and a decrease in CD4+ T cells were observed, indicating immune dysregulation. Significant metabolic diversity within T cell subpopulations led to the classification of patients with T2DM into three distinct subtypes (A-C), with HC grouped as D. Enhanced intercellular communication, particularly through the MHC-I pathway, was evident in T2DM subtypes. Machine learning models effectively classified T2DM subtypes based on metabolic signatures, achieving an AUC > 0.84. Analysis of TF activity identified pivotal regulators, including NF-kB, STAT3, and FOXO1, associated with immune and metabolic disturbances in T2DM. Drug enrichment analysis highlighted potential therapeutic agents targeting these TFs and related pathways, including Suloctidil, Chlorpropamide, and other compounds modulating inflammatory and metabolic pathways.

**Conclusion:**

This study underscores significant immunometabolic dysfunction in T2DM, characterized by alterations in immune cell composition, metabolic pathways, and intercellular communication. The identification of critical TFs and the development of drug enrichment profiles highlight the potential for personalized therapeutic strategies, emphasizing the need for integrated immunological and metabolic approaches in T2DM management.

## Introduction

Type 2 Diabetes Mellitus (T2DM) represents a growing global health crisis, with prevalence rates increasing rapidly. The International Diabetes Federation estimates that 537 million adults were living with diabetes in 2021, a number projected to rise to 643 million by 2030 and 783 million by 2045 (1). T2DM accounts for 90-95% of all diabetes cases and is a leading contributor to morbidity and mortality, with associated complications such as cardiovascular disease, neuropathy, nephropathy, and retinopathy (2) The rising incidence of T2DM is driven by a combination of genetic predisposition and lifestyle factors, including obesity, sedentary behavior, and poor dietary habits (3).

Beyond its metabolic consequences, T2DM is increasingly recognized for its significant immunological components, characterized by chronic low-grade inflammation and immune dysregulation (4). Peripheral blood mononuclear cells (PBMCs), including T cells and monocytes, play pivotal roles in the inflammatory processes of T2DM (5). Alterations in immune cell populations have been documented in patients with T2DM, with changes observed in the proportions and functions of various immune cell subsets (6).

T cells and monocytes are particularly implicated in T2DM pathogenesis through their contribution to systemic inflammation and insulin resistance (7). Chronic activation of these immune cells results in the secretion of pro-inflammatory cytokines, which disrupt insulin signaling pathways (8). However, the precise mechanisms by which these immune cells contribute to T2DM, particularly at the single-cell level, remain poorly understood.

Recent advancements in single-cell RNA sequencing (scRNA-seq) have enabled high-resolution analysis of cellular heterogeneity, facilitating the characterization of individual cell types and states within complex tissues (9). This technology offers a unique opportunity to explore the immunological landscapes of PBMCs in T2DM at an unprecedented level of detail. By analyzing gene expression profiles at the single-cell level, it is possible to identify specific cellular subpopulations and uncover new insights into the disease mechanisms.

Metabolic reprogramming of immune cells is a critical aspect of their activation and function (10). In the context of T2DM, metabolic disturbances can influence immune cell behavior, contributing to disease progression (10). Metabolic reprogramming in T cells and monocytes plays a pivotal role in the pathogenesis of T2DM (11). Immune cells, like T cells and monocytes, undergo metabolic shifts in T2DM, which affect their activation and function, thereby exacerbating chronic inflammation and insulin resistance (11). These metabolic alterations can promote the secretion of pro-inflammatory cytokines, further driving disease progression (12). Understanding how metabolic reprogramming influences immune cell behavior could identify novel therapeutic targets for T2DM.

Furthermore, cell-cell communication, mediated by signaling pathways and cytokines, is essential for orchestrating immune responses (13). Dysregulation of these communication networks can intensify inflammation and insulin resistance in T2DM (14). Investigating intercellular signaling dynamics may reveal potential therapeutic targets for modulating immune responses.

In this study, publicly available scRNA-seq data were used to analyze PBMCs from patients with T2DM and healthy controls (HC). This study aimed to characterize the immune cell composition, metabolic heterogeneity, and cell-cell communication networks at the single-cell level. Additionally, advanced machine learning models were employed to classify T2DM subtypes based on metabolic signatures. The findings offer comprehensive insights into the immunometabolic alterations in T2DM, providing a foundation for the development of personalized therapeutic strategies.

## Methods

### Data collection

The sequencing data used in this study are publicly available from the Gene Expression Omnibus (GEO) database. scRNA-seq data for PBMCs from 11 HC individuals (GSE244515) (15) and 37 patients diagnosed with T2DM (GSE268210) (16) were utilized.

### Single-cell RNA sequencing alignment and quality control

All single-cell read counts were analyzed using the Seurat package (v5.0.1) in R (v4.3.1), converting each dataset into individual Seurat objects. Data filtering was performed based on unique molecular identifiers (UMIs) and the number of detected genes (17). Specifically, cells with between 500 and 3,500 detected genes, and those expressed in at least five cells, were retained. Cells exhibiting mitochondrial gene expression greater than 5% were excluded to ensure data quality. Following filtering, data normalization was carried out using Seurat’s NormalizeData function, and highly variable genes were identified using the FindVariableFeatures function.

### Integration of scRNA-seq data from multiple datasets

To integrate scRNA-seq data from multiple datasets, the Harmony package was employed, focusing on highly variable genes. This integration enabled subsequent dimensionality reduction and clustering analyses, correcting for batch effects and other technical variations across datasets.

### Dimensionality reduction and major cell type annotation

**Figure 1:**
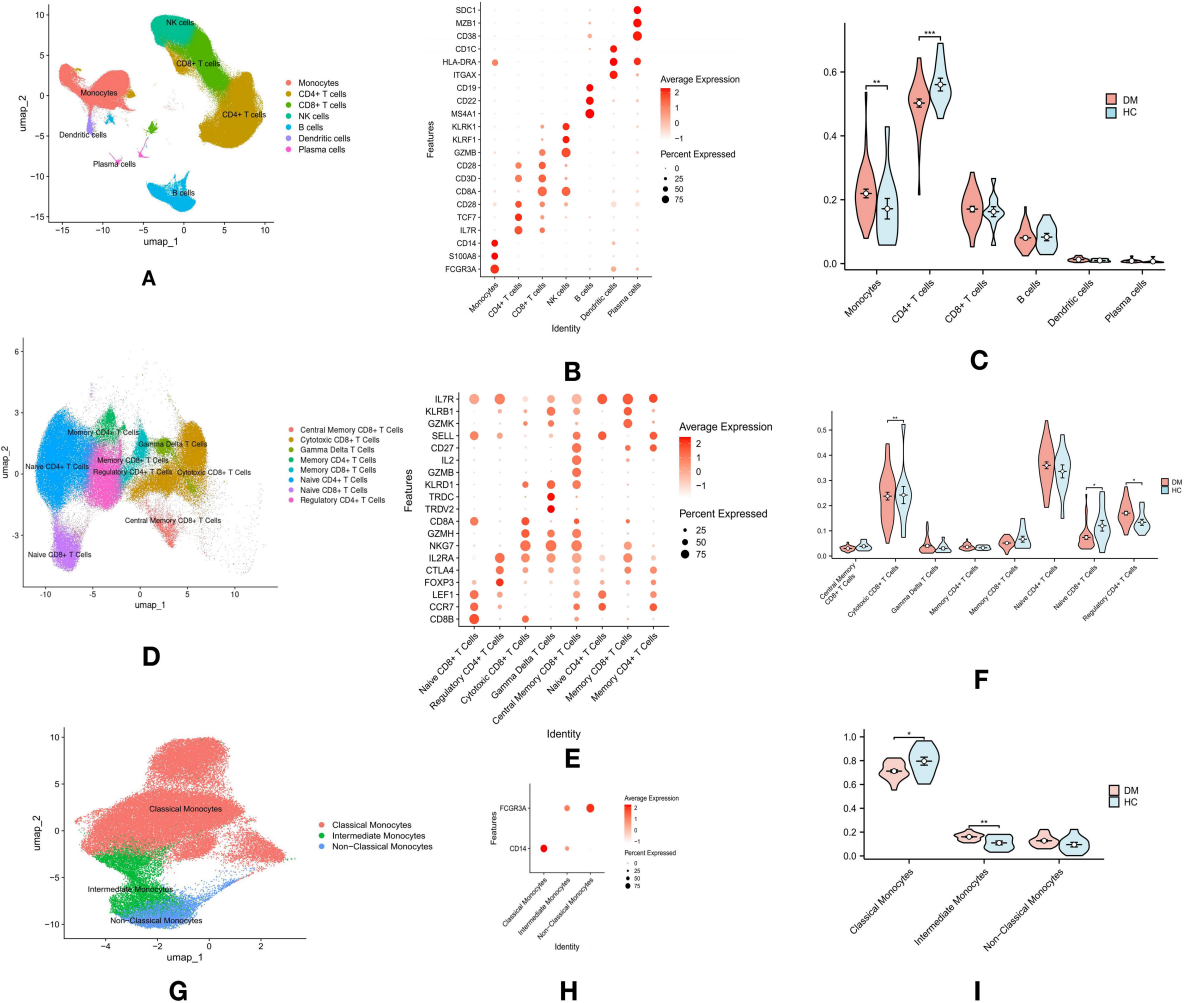
Overview of Immune Cell Profiling in T2DM and Healthy Control (HC) Groups: **(A)** Single-cell RNA sequencing and clustering analysis identified seven major immune cell types in PBMCs from both T2DM and HC groups. **(B)** The top three marker genes for each of the seven major immune cell types in PBMCs. **(C)** Violin plots comparing the proportions of these seven immune cell types in PBMCs across T2DM and HC groups. **(D)** Dimensional reduction analysis of T cell clusters, revealing eight distinct T cell subtypes. **(E)** The top three marker genes for each of the eight T cell subtypes. **(F)** Violin plots comparing the proportions of the eight T cell subtypes in PBMCs. **(G)** Monocyte subpopulation analysis identified three distinct subtypes: classical, intermediate, and non-classical monocytes. **(H)** Expression of marker genes (CD14 and FCG3RA) used for classifying monocyte subpopulations. **(I)** Violin plots comparing the proportions of the three monocyte subtypes. p-values are indicated as follows: *p ≤ 0.05, **p ≤ 0.01, and ***p ≤ 0.001.

For the PBMC dataset, clustering resolution was set to 0.5. Principal component analysis (PCA) was used for dimensionality reduction, followed by Uniform Manifold Approximation and Projection (UMAP) for visualization. Clusters were identified and annotated based on known cell type markers, as shown in **Figures 1B, 1E**, and **1H**

### Differential gene expression and pathway analysis

Differential gene expression analysis was conducted using the FindMarkers function of the Seurat package, employing the Wilcoxon rank-sum test. Genes were considered differentially expressed if detected in at least 25% of cells (min.pct = 0.25) and had an adjusted p-value below 0.05 after Bonferroni correction. Significant differentially expressed genes (DEGs) were subjected to Gene Ontology (GO) term and Kyoto Encyclopedia of Genes and Genomes (KEGG) pathway enrichment analyses using the clusterProfiler package (v3.12.0) (18). Drug enrichment analysis was performed using the Drug-Gene Interaction Database (DGIdb) as the reference, selecting enriched drugs with an adjusted p-value threshold of P < 0.05 after multiple testing correction.

### Gene set variation analysis

Gene Set Variation Analysis (GSVA) was employed to assess pathway activity across single cells using 42 KEGG pathways as predefined gene sets. The GSVA method was implemented with the GSVA package in R, specifying appropriate gene set indices and kernel-based distribution functions (kcdf). To optimize computational efficiency, parallel processing was utilized, with parameter adjustments based on available processor cores. This approach allowed for scalable analysis, reduced processing time, and preserved result integrity. GSVA provided pathway activity scores for each cell, enabling the exploration of pathway heterogeneity and functional states within the single-cell populations.

### Calculation of transcription factor activity

To assess transcription factor (TF) activity, the DoRothEA package was used to retrieve human regulon data, selecting regulons with confidence levels A, B, and C (19). TF activity scores were calculated using the VIPER method, with normalization performed *via* the “scale” method and a minimum regulon size of 4. These scores were stored in the “dorothea” assay of the Seurat object. Dimensionality reduction was conducted using PCA, followed by clustering with the top 10 principal components, and UMAP was applied for cluster visualization. Differential TF activity between clusters was evaluated using Seurat’s FindAllMarkers function, with significant TFs identified based on log fold change and expression percentage. The VIPER activity scores were summarized by cell type, and the three most variable TFs across cell types were identified. These TFs were visualized in a heatmap, with color intensities reflecting TF activity.

### Unsupervised clustering (consensus clustering)

To classify patients with T2DM based on T cell metabolic patterns, consensus clustering was applied, a robust and reproducible method that aggregates multiple clustering results to enhance stability and reliability using the ConsensusClusterPlus package. Initially, the mean GSVA scores for the 42 pathways were calculated for each sample. Consensus clustering mitigates inherent variability in individual clustering runs by repeatedly subsampling the data and aggregating clustering results, ensuring the identification of consistent and biologically meaningful clusters.

The optimal number of clusters (k) was determined by calculating the incremental area, which measures changes in the cumulative distribution function (CDF) curve area between consecutive k values. The incremental area quantifies improvements in cluster stability as the number of clusters increases. A significant drop in the incremental area suggests that additional clusters contribute minimally to cluster stability, aiding in the selection of the optimal k. Consensus clustering was performed across a range of k values (from k = 2 to k = 9), and incremental area plots were generated to visualize changes in the CDF curve areas. Using the “elbow method,” where the k value at which the incremental area plateaus is selected (indicating diminishing returns from adding more clusters), we identified k = 4 as the optimal number. From k = 4 onward, the reduction in incremental area was significantly less, indicating that k = 4 struck a balance between minimizing the metric and maintaining manageable cluster numbers. Clustering at k = 4 was subsequently visualized using heatmaps and PCA plots.

### Cell communication and signaling pathways

Cell communication analysis was performed using the CellChat package in R with default parameters (20). The pathways mediating cell communication between three T cell subtypes and monocytes were analyzed independently, utilizing the human CellChatDB as a reference. The rankNet function was modified to output scaled contribution values for each pathway within each subtype. Differences in the strength of cell communication pathways between the T cell subtypes and monocytes were compared and visualized with bar charts generated by ggplot2. Additionally, specific signaling patterns for each pathway within each subtype were illustrated using the netVisual_bubble function.

### Machine learning algorithms

An integrated machine learning model incorporating multiple algorithms was developed to enhance predictive accuracy. A comprehensive dataset of 196,623 T cells was divided into a training set (70%) and a test set (30%). A total of 75 different combinations of machine learning models were evaluated. Independent predictive models included Support Vector Machines (SVM) and Ridge regression. Boosting methods such as glmBoost, Elastic Net (Enet) with varying alpha values, and Gradient Boosting Machines (GBM) were sequentially applied to correct errors from previous models. Stepwise regression (Stepglm), utilizing forward, backward, or both selection criteria, was combined with models like Ridge, Enet, and Lasso to optimize predictive performance. Additional models, including XGBoost, Linear Discriminant Analysis (LDA), Random Forest (RF), and Naive Bayes, were integrated to leverage the unique strengths of each algorithm in different scenarios.

For multiclass classification adjustments, both one-vs-rest (OvR) and multinomial classification approaches were employed. The OvR strategy decomposes the multiclass problem into multiple binary classifiers, each distinguishing one class from all others. This method was applied to SVM and Logistic Regression algorithms to establish binary decision boundaries within a multiclass framework. Multinomial classification methods, such as GBM and RF, handle all classes simultaneously within a single model, allowing for direct modeling of class probabilities. These algorithms natively support multinomial classification, enabling the simultaneous prediction of multiple classes without decomposing them into separate binary tasks. The choice of methods was guided by the algorithm’s native support for multiclass classification and empirical performance during model tuning.

At the patient level, individuals were classified based on the distribution of cell subtypes within their samples. If the majority of a patient’s cells were assigned to a specific subtype, the patient was classified into that subtype. This strategy enabled the extension of single-cell classification to predict subtypes at the patient level.

Models were configured to identify the one with the highest average concordance index (C-index) across all validation datasets. The accuracy of the resulting risk scores was validated by calculating the area under the curve (AUC) using the “timeROC” package.

### Statistical analysis

All statistical analyses were performed using R software (v4.3.1), and visualizations were generated through R Studio. The selection of statistical tests was determined by the data distribution and characteristics. For normally distributed data, Student’s t-test was used to compare means between two groups. For non-normally distributed data, the Wilcoxon rank-sum test was applied for two-group comparisons, and the Kruskal-Wallis test was utilized for comparisons across multiple groups. P-values > 0.05 were considered not statistically significant and were marked as “ns.” P-values ≤ 0.05 were considered statistically significant, with the following indications: * p ≤ 0.05, ** p ≤ 0.01, *** p ≤ 0.001, and **** p ≤ 0.0001.

## Results

### Significant increase in monocytes and decrease in CD4+ T cells in patients with T2DM

Single-cell sequencing data of PBMCs from 11 HC (GSE244515) and 37 patients with T2DM (GSE268210) were obtained from the GEO database. Cluster analysis revealed seven major immune cell types, annotated by specific marker genes: CD4+ T cells (CD3D, IL7R), CD8+ T cells (CD3D, CD8B), NK cells (KLRF1), B cells (MS4A1), monocytes (CD14, FCG3RA), dendritic cells (ITGAX, CD1C), and plasma cells (SDC1, MZB1) (**Figure 1A**). Cell types were annotated using established marker genes, a method validated in prior studies (**Figure 1B**). Rigorous marker selection and clustering methods were applied to ensure accurate and consistent categorization of cell types within the datasets. Proportions of immune cell types between T2DM and HC groups were compared using the Wilcoxon test. The analysis revealed a significant increase in monocyte proportions (p < 0.01) and a decrease in CD4+ T cells (p < 0.01) in the T2DM group compared to the HC group, while no significant differences were observed in CD8+ T cells, B cells, dendritic cells, or plasma cells (**Figure 1C**).

### Altered proportions of T cell subtypes in patients with T2DM

Dimensional reduction and cluster analysis of T cells based on gene expression profiles identified eight distinct subtypes: Central Memory CD8+ T cells (IL7R, CD27, SELL), Cytotoxic CD8+ T cells (CD8A, GZMH, NKG7), Gamma Delta T cells (TRDC, TRDV2), Memory CD4+ T cells (IL7R, CD27), Memory CD8+ T cells (IL7R, CD27), Naive CD4+ T cells (LEF1, SELL, CCR7), Naive CD8+ T cells (CD8A, LEF1, CCR7), and Regulatory CD4+ T cells (FOXP3) (**Figure 1D, E**). SELL expression was utilized to distinguish between Central Memory and Memory CD8+ T cells. Differences in T cell subtype proportions between T2DM and HC groups were assessed using the Wilcoxon test. Significant increases in the proportions of Cytotoxic CD8+ T cells (p < 0.01) and Naive CD8+ T cells (p < 0.05) were observed in the T2DM group, alongside a significant reduction in Regulatory CD4+ T cells (p < 0.05). No significant differences were found in Central Memory CD8+ T cells, Gamma Delta T cells, Memory CD4+ T cells, Memory CD8+ T cells, or Naive CD4+ T cells (**Figure 1F**).

### Changes in monocyte subpopulations in patients with T2DM

The interaction between monocytes and T cells plays a critical role in the inflammatory mechanisms driving T2DM progression (21). Monocytes modulate T cell responses and are central to the immune dysregulation observed in T2DM (22). Further analysis of monocytes revealed three subgroups: classical monocytes, non-classical monocytes, and intermediate monocytes (**Figure 1G**). Classical monocytes were defined by CD14 expression, non-classical by CD16, and intermediate by both CD14 and CD16 (**Figure 1H**). The Wilcoxon test revealed a significant increase in intermediate monocytes (p < 0.01) and a decrease in classical monocytes (p < 0.05) in the T2DM group compared to the HC group (**Figure 1I**).

### Metabolic heterogeneity in T cell subpopulations in T2DM

**Figure 2:**
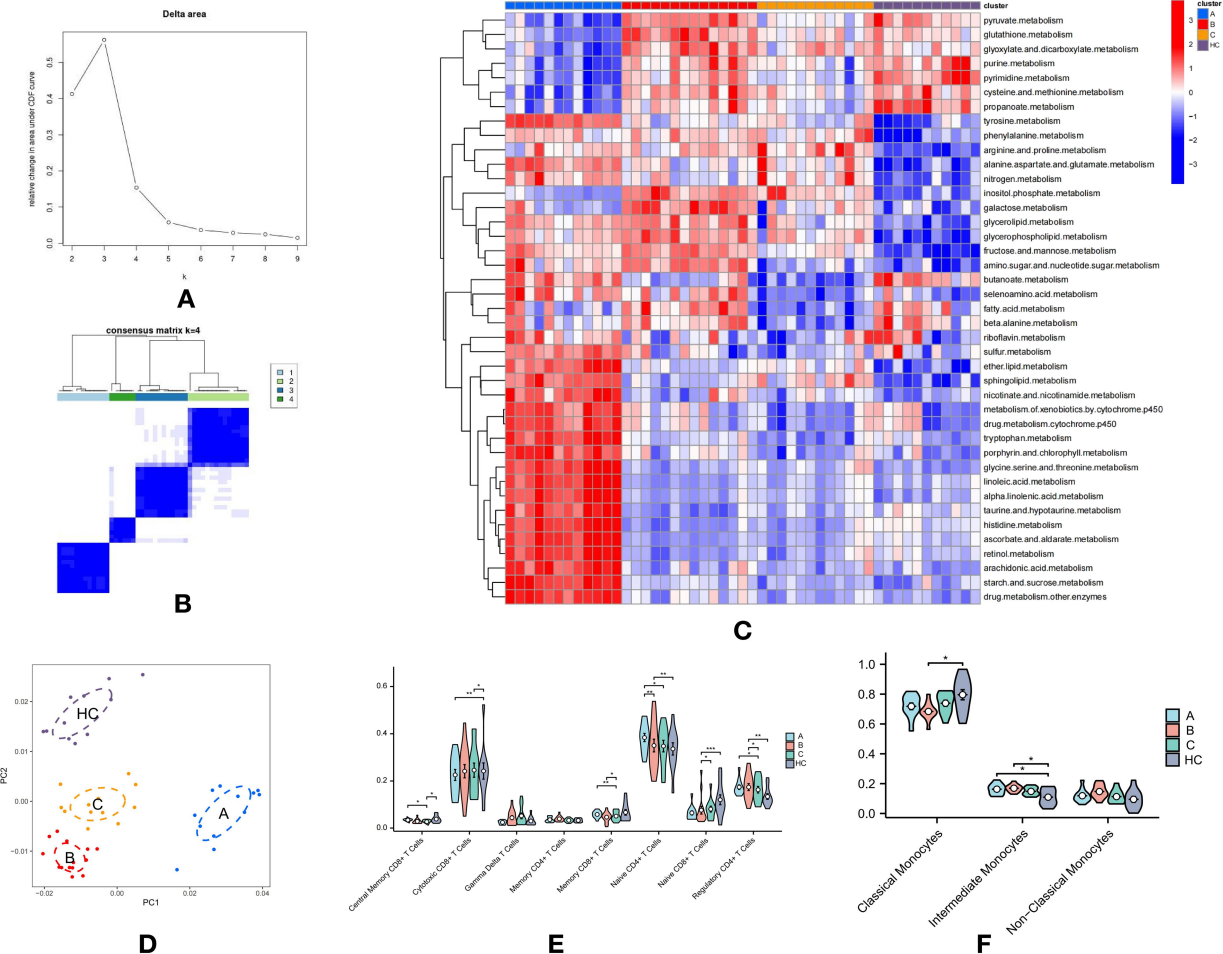
Metabolic Heterogeneity in T Cell Subpopulations and Immunological Differences in T2DM: **(A)** Delta area plot showing k values from 2 to 9 used for selecting the optimal k in consensus clustering. **(B)** Consensus clustering heatmap of metabolic pathway scores, dividing samples into four distinct groups. Groups A-C consist of T2DM samples, while Group D represents healthy control (HC) samples. **(C)** Clustering heatmap displaying the expression levels of 42 metabolic-related pathways in T cell subpopulations across the four groups, emphasizing differences in metabolic activity. **(D)** PCA plot demonstrating clear separation between the HC group **(D)** and T2DM groups **(A-C)**. **(E)** Violin plots showing the proportions of eight T cell subtypes across the four groups, with significant differences observed between groups. **(F)** Violin plots illustrating the proportions of three monocyte subtypes across the four groups, highlighting further immune profile differences. p-values are indicated as follows: *p ≤ 0.05, **p ≤ 0.01, and ***p ≤ 0.001.

To investigate the metabolic heterogeneity within T cell subpopulations in T2DM, each cell within these subpopulations was scored for 42 metabolic-related pathways from the KEGG database using GSVA. Unsupervised consensus clustering, based on the mean pathway values for each sample, was performed. The optimal number of clusters (k = 4) was determined using the delta area value and the “elbow method,” partitioning the samples into four groups (**Figure 2A**). The clustering heatmap clearly distinguished the samples into four groups, with T2DM samples assigned to groups A-C and HC samples grouped in D (**Figure 2B**). This segregation was further validated by the PCA plot, which highlighted a distinct separation between group D (HC) and groups A-C (**Figure 2D**). Specifically, group A included 12 patients, group B included 14 patients, group C included 12 patients, and group D contained 11 HC.

Group A exhibited elevated expression across various metabolic pathways, including sulfur, ether lipid, and sphingolipid metabolism; nicotinate and nicotinamide metabolism; xenobiotic and drug metabolism by cytochrome P450; tryptophan, porphyrin, and chlorophyll metabolism; glycine, serine, and threonine metabolism; linoleic and alpha-linolenic acid metabolism; taurine and hypotaurine metabolism; histidine metabolism; ascorbate and aldarate metabolism; retinol metabolism; arachidonic acid metabolism; and starch and sucrose metabolism, among others (**Figure 2C**). This broad metabolic profile, encompassing lipid, amino acid, and complex carbohydrate pathways, suggests an adaptive metabolic response in T cells within Group A.

Group B was distinguished by high expression in pathways such as pyruvate, glutathione, glyoxylate and dicarboxylate, purine, pyrimidine, cysteine and methionine, nitrogen, inositol phosphate, galactose, glycerolipid, glycerophospholipid, fructose and mannose, and amino sugar and nucleotide sugar metabolism (**Figure 2C**). This unique metabolic signature suggests a specific adaptation in Group B, likely reflecting a distinct functional or activation state compared to other groups.

Group C shared a metabolic profile with Group B, marked by high expression in nitrogen, inositol phosphate, galactose, glycerolipid, glycerophospholipid, fructose and mannose, and amino sugar and nucleotide sugar metabolism (**Figure 2C**). However, the metabolic reprogramming in Group C appeared more targeted or restricted, suggesting a more specific metabolic shift in the T cells.

Group D, representing the HC group, displayed strong expression in pyruvate, glutathione, glyoxylate and dicarboxylate, purine, pyrimidine, cysteine and methionine, propanoate, butanoate, fatty acid, and beta-alanine metabolism (**Figure 2C**). This metabolic profile aligns with basic cellular metabolism and energy homeostasis, contrasting with the altered metabolic states observed in the T2DM groups.

### Immunological differences between T2DM subtypes and HC group

The Kruskal-Wallis test was performed to examine immunological differences in T cell and monocyte subtypes across the groups, revealing significant alterations indicative of substantial immune modulation in T2DM. Notably, Groups A and HC displayed increased proportions of Central Memory CD8+ T Cells, essential for long-term immune memory, suggesting potential immune adaptation or ongoing immune responses. A significant reduction in Cytotoxic CD8+ T Cells was observed in Groups A and C compared to the HC group, indicating an impaired cytotoxic response critical for targeting infected or dysfunctional cells (**Figure 2E**).

Additionally, a decrease in Memory and Naive CD8+ T Cells in Group C suggests a compromised adaptive immune response, essential for effective long-term immunity. The reduction in Regulatory CD4+ T Cells, especially in Group C, suggests diminished regulatory function, potentially contributing to unchecked immune responses and inflammation characteristic of chronic conditions like T2DM (**Figure 2E**).

Moreover, a significant reduction in classical monocytes in Group B (P < 0.05) was observed, while proportions of intermediate monocytes were significantly increased in Groups A and B (P < 0.05) compared to the HC group (**Figure 2F**).

These findings underscore the intricate interplay between metabolic and immune shifts in T2DM, illustrating how metabolic disturbances may impact immune function and potentially exacerbate the disease. The distinct metabolic profiles observed in T2DM subgroups suggest that targeted metabolic or immunomodulatory therapies could be tailored to address specific dysregulations in these patients.

### The communication between T-cells and monocytes in type 2 diabetes

The communication between T-cells and monocytes in T2DM plays a critical role, profoundly influencing immune regulation, inflammation, and autoimmunity, all pivotal in the disease’s progression and management. Understanding these interactions offers insights into how immune dysregulation contributes to chronic inflammation and insulin resistance in T2DM.

**Figure 3:**
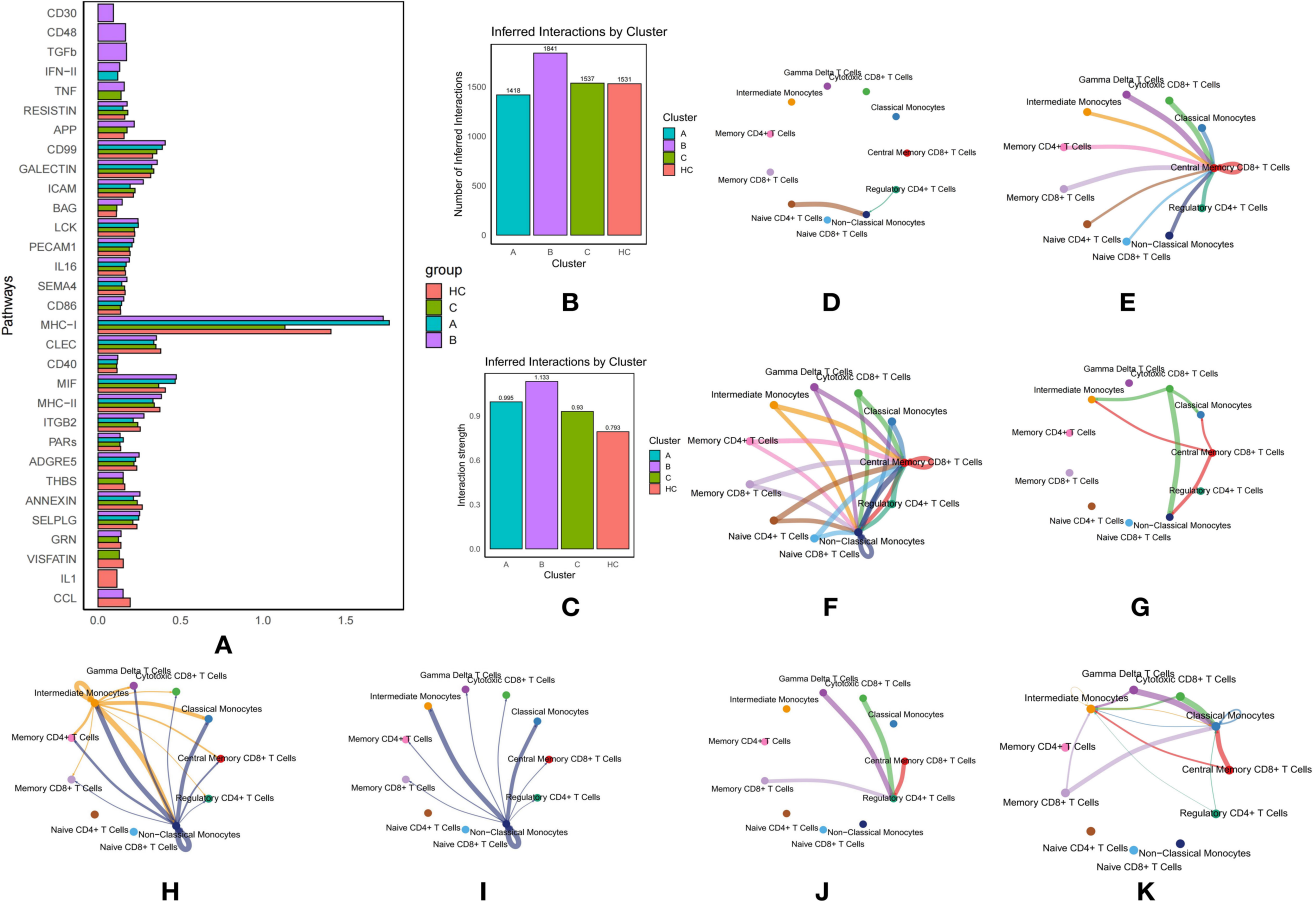
Communication Between T Cells and Monocytes in Type 2 Diabetes Mellitus (T2DM): **(A)** Comparison of pathway activity across T2DM subtypes and healthy controls (HC). **(B)** Number of inferred interactions by cluster. **(C)** Interaction strength by cluster. **(D)** Network diagram illustrating key pathway-mediated interactions between T cell and monocyte subtypes for the CD30 pathway. **(E)** Network diagram illustrating key pathway-mediated interactions between T cell and monocyte subtypes for the TGF-β pathway. **(F)** Network diagram illustrating key pathway-mediated interactions between T cell and monocyte subtypes for the CD48 pathway. **(G)** Network diagram illustrating key pathway-mediated interactions between T cell and monocyte subtypes for the IFN-γ pathway. **(H)** Network diagram illustrating TNF pathway interactions in T2DM subtype B. **(I)** Network diagram illustrating TNF pathway interactions in T2DM subtype C. **(J)** Network diagram illustrating CCL pathway interactions in T2DM subtype B. **(K)** Network diagram illustrating CCL pathway interactions in HC.

CellChat was employed to analyze communication differences between T-cells and monocytes across three T2DM subtypes and an HC group (**Supplementary Tables 1**–**4**). Regarding the number of inferred interactions, Subtypes A, B, C, and HC had 1418, 1841, 1537, and 1531 interactions, respectively (**Figure 3B**). Interaction strength values were 0.995 for Subtype A, 1.133 for Subtype B, 0.93 for Subtype C, and 0.793 for HC (**Figure 3C**). These results highlight variability in communication intensity and complexity across diabetic subtypes compared to HC (**Figure 3A**), indicating stronger cellular interactions in patients with T2DM, suggesting an enhanced immune response in diabetic conditions.

### Intensive pathway mediation in subtype B

In Subtype B of T2DM, multiple pathways actively mediate communication between immune cells. The CD30 pathway facilitates interactions from Naive CD4+ T Cells and Regulatory CD4+ T Cells to Non-Classical Monocytes, which serve as receptors (**Figure 3D**). This pathway is pivotal as it involves T cells that are essential for maintaining immune tolerance and preventing autoimmune responses while interacting with monocytes that play a central role in inflammation. Activation of this pathway in Subtype B suggests a specific immune regulatory mechanism that could significantly impact the inflammatory environment characteristic of T2DM.

Similarly, the CD48 pathway orchestrates communication between three monocyte subtypes and various T-cell subtypes to Central Memory CD8+ T Cells, also functioning as receptors, and extends this interaction to include Non-Classical Monocytes (**Figure 3F**). This pathway underscores a robust exchange of signals, enhancing immune memory and responsiveness, which is essential for managing recurrent or chronic antigen exposure in T2DM.

Additionally, the Transforming Growth Factor Beta (TGF-β) pathway mediates interactions from multiple T-cell and monocyte subtypes to Central Memory CD8+ T Cells (**Figure 3E**). TGF-β, a key cytokine in regulating immune responses, cell growth, and inflammation, suggests a dual role in promoting immune homeostasis and potentially contributing to immune tolerance in T2DM.

The Interferon Type II (IFN-II) pathway is prominently active in Subtypes B and C, facilitating signals from Cytotoxic CD8+ T Cells to Classical and Non-Classical Monocytes, and from Central Memory CD8+ T Cells to Intermediate Monocytes (**Figure 3G**). The engagement of this pathway highlights an active antiviral and antitumor response, which may be dysregulated in T2DM, contributing to altered immune cell activation and cytokine production and influencing disease progression.

The extensive involvement of these pathways in Subtype B reveals a complex and distinct immune modulation pattern that may significantly influence the clinical manifestations and progression of T2DM. The differential activation of these pathways underscores the intricate interplay between immune cells in diabetes, providing a foundation for the development of targeted therapeutic strategies.

### TNF and CCL pathway involvement

The Tumor Necrosis Factor (TNF) pathway was particularly active in Subtype B, mediating communication from Intermediate Monocytes to other monocyte and T-cell subtypes (acting as receptors), and from Non-Classical Monocytes to various monocyte and T-cell subtypes (acting as receptors) (**Figure 3H**). In contrast, in Subtype C, the TNF pathway exclusively mediated communication from Non-Classical Monocytes to other monocyte and T-cell subtypes (as receptors), suggesting its involvement in promoting inflammatory processes that may exacerbate diabetes complications (**Figure 3I**).

The CCL pathway in Subtype B specifically mediated interactions with Regulatory CD4+ T Cells as receptors and Gamma Delta T Cells, Cytotoxic CD8+ T Cells, Central Memory CD8+ T Cells, and Memory CD8+ T Cells as ligands (**Figure 3J**). In the HC group, the CCL pathway significantly mediated communication with Classical Monocytes and Intermediate Monocytes as receptors (**Figure 3K**). The differential involvement of this pathway highlights its potential role in modulating immune responses differently in diabetic patients versus healthy individuals.

### MHC-I pathway dominance

The Major Histocompatibility Complex Class I (MHC-I) pathway contributed extensively across all three subtypes, mediating nearly all communication between T-cell and monocyte subtypes (**Figure 3A**). Subtype A exhibited the highest activity, followed by HC, with Subtype C showing the least. This underscores the pivotal role of antigen presentation in T2DM, which could influence autoimmune responses and overall immune function in these patients.

### VISFATIN pathway specificity

The VISFATIN pathway, uniquely present in Subtypes C and HC, was involved exclusively in mediating communication among T-cell subtypes, without interactions between T-cells and monocytes. This selective engagement suggests a distinct metabolic or inflammatory state inherent to these subtypes and indicates that VISFATIN may play a role in unique disease progression pathways or therapeutic resistance mechanisms in T2DM. The focused activity of VISFATIN offers insights into subtype-specific immune functions, potentially guiding more personalized treatment approaches.

### Additional pathways mediated T-cell and monocyte communication

Further analysis revealed additional pathways—GRN, SELPLG, ANNEXIN, THBS, ADGRE5, PARs, ITGB2, MHC-II, MIF, CD40, CLEC, CD86, SEMA4, IL16, PECAM1, LCK, BAG, ICAM, GALECTIN, CD99, APP, and RESISTIN—that mediate communication across various T-cell and monocyte subtypes (**Figure 3A**). These pathways are involved in a range of regulatory and signaling processes, such as adhesion, immune response modulation, and inflammation. Their involvement across multiple subtypes highlights the complexity and dynamic nature of cellular communication in T2DM, emphasizing the potential for targeted therapeutic interventions based on these specific molecular interactions.

### Analysis of transcription factor activity across diabetes subtypes

**Figure 4:**
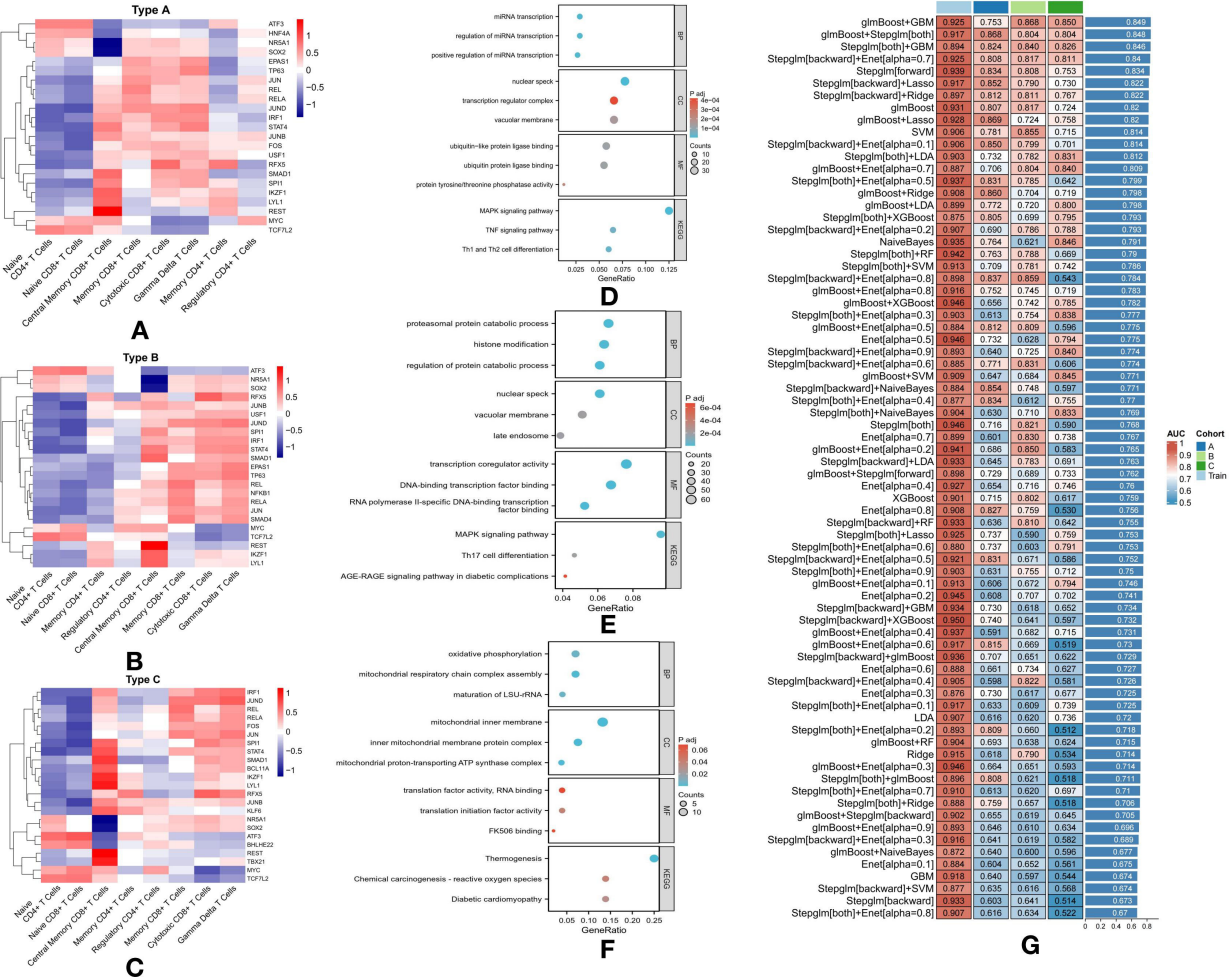
Transcription Factor Activation and Pathway Enrichment Analysis in T2DM Subtypes with Machine Learning Classification: **(A)** Heatmap showing logFC of the top three transcription factors in each cell type of Subtype A. **(B)** Heatmap showing logFC of the top three transcription factors in each cell type of Subtype B. **(C)** Heatmap showing logFC of the top three transcription factors in each cell type of Subtype C. **(D)** Pathway enrichment analysis of 436 differentially expressed genes in Subtype A. **(E)** Pathway enrichment analysis of 845 differentially expressed genes in Subtype B. **(F)** Pathway enrichment analysis of 122 differentially expressed genes in Subtype C. **(G)** Performance of various machine learning models for classifying T2DM subtypes based on T-cell metabolic characteristics.

We also analyzed TF activity across the three T2DM subtypes, identifying 126 active TFs. Key examples include IRF1, GATA6, SPI1, EPAS1, NFKB2, and STAT5B, which are involved in immune response, cell differentiation, and metabolic regulation, all of which are critical in diabetes pathogenesis. A heatmap was generated to visualize the top three TFs for each cell type across the subtypes (**Figures 4A–C**).

### Subtype A: activation of transcription factors in immune cells

In Subtype A, TFs were notably active in Central Memory CD8+ T Cells, Memory CD8+ T Cells, Cytotoxic CD8+ T Cells, and Gamma Delta T Cells, indicating an enhanced immune response (**Figure 4A**). Of particular interest, HNF4A was uniquely active in Naive CD4+ T Cells and Naive CD8+ T Cells, suggesting a role in early immune cell activation. EPAS1, a key factor involved in oxygen sensing and cellular stress responses, was active in Memory CD8+ T Cells, Cytotoxic CD8+ T Cells, and Gamma Delta T Cells, highlighting its involvement in regulating immune cell function during inflammatory or stress-induced conditions (**Figure 4A**).

### Subtype B: immune modulation and inflammation

In Subtype B, similar TF activity was observed in Central Memory CD8+ T Cells, Memory CD8+ T Cells, Cytotoxic CD8+ T Cells, and Gamma Delta T Cells, with additional unique findings (**Figure 4B**). NFKB1, known for its role in immune modulation and inflammation, was specifically active in Regulatory CD4+ T Cells, suggesting its contribution to immune tolerance and the prevention of autoimmunity in this subtype. Additionally, SMAD4, a central player in the TGF-β signaling pathway, was active across several T cell types, indicating its role in immune response regulation and tissue remodeling in Subtype B (**Figure 4B**).

### Subtype C: strong immune activation and differentiation

In Subtype C, TFs such as SPI1, STAT4, SMAD1, BCL11A, IKZF1, LYL1, REST, and TBX21 were highly active in Central Memory CD8+ T Cells, suggesting robust immune activation and differentiation (**Figure 4C**). BCL11A, active in Central Memory CD8+ T Cells, Cytotoxic CD8+ T Cells, and Gamma Delta T Cells, plays a critical role in these cell types. Moreover, BHLHE22, active in Naive CD4+ T Cells and Naive CD8+ T Cells, may regulate early-stage immune responses (**Figure 4C**). KLF6, active in Central Memory CD8+ T Cells and Memory CD4+ T Cells, likely governs immune cell differentiation and survival. Lastly, TBX21, essential for T cell differentiation and function, was active in Central Memory CD8+ T Cells, underscoring its role in shaping long-term immune responses in this subtype (**Figure 4C**).

### Differential gene expression in subtype A

For Subtype A, further analysis revealed 436 DEGs, highlighting significant involvement in pathways related to microRNA (miRNA) transcription and immune system regulation (**Figure 4D**). The enrichment of miRNA-related pathways, such as positive regulation of miRNA transcription, regulation of miRNA transcription, and miRNA transcription itself, suggests that miRNAs play a critical role in controlling gene expression that modulates T-cell function and overall immune responses (**Figure 4D**). This subtype also exhibited significant enrichment in immune-related pathways, including the MAPK signaling pathway, TNF signaling pathway, and Th1/Th2 cell differentiation (**Figure 4D**). These pathways are pivotal in mediating immune responses and likely contribute to the inflammatory state observed in diabetes and its associated complications in Subtype A. The presence of these pathways highlights the intricate interplay between genetic regulation and immune responses, offering potential insights for developing targeted therapeutic strategies for this subtype.

### Differential gene expression in subtype B

Subtype B, distinguished by 845 DEGs, is characterized by a broad range of enriched pathways primarily related to protein metabolism and modification (**Figure 4E**). Pathways such as the regulation of protein catabolic processes, proteasomal protein catabolism, and histone modification highlight an increased focus on protein turnover and post-translational modifications, both critical for cellular function and signaling. Immune-related pathways, including the MAPK signaling pathway, AGE-RAGE signaling in diabetic complications, and Th17 cell differentiation, are also prominently represented (**Figure 4E**). The AGE-RAGE pathway is particularly notable for linking metabolic dysregulation to inflammatory responses, a hallmark of diabetes-related complications (**Figure 4E**). Furthermore, the Th17 differentiation pathway suggests the involvement of a specific T-cell subset known for its role in inflammation and autoimmunity, potentially contributing to the pathophysiological complexity observed in Subtype B (**Figure 4E**).

### Differential gene expression in subtype C

In Subtype C, the 122 DEGs are significantly enriched in pathways related to metabolic processes, with a particular focus on oxidative phosphorylation, a key energy production mechanism in cells (**Figure 4F**). The inclusion of pathways such as chemical carcinogenesis—reactive oxygen species and diabetic cardiomyopathy points to an increased susceptibility to oxidative stress and its associated cardiac complications, common challenges in diabetes management (**Figure 4F**). The prominence of oxidative phosphorylation suggests altered metabolic function that may exacerbate energy deficits in diabetic cells, potentially driving cellular dysfunction and cardiomyopathy progression (**Figure 4F**). The emphasis on metabolic and oxidative stress pathways in this subtype underscores the importance of metabolic control and highlights potential therapeutic targets for addressing these specific challenges.

### Advanced machine learning models for subtype classification

This study further developed machine learning models to differentiate T2DM subtypes based on the metabolic characteristics of T-cells, derived from the GSVA results of KEGG metabolic pathways for each individual cell. A total of 75 model combinations were evaluated, with particular emphasis on high-performing models such as glmBoost+GBM, glmBoost+Stepglm (both combinations), Stepglm+GBM, and Stepglm (backward)+Enet [alpha = 0.7] (**Figure 4G**).

GlmBoost, or Generalized Linear Model Boosting, enhances prediction accuracy by combining multiple weak models, typically linear, into a stronger predictive ensemble. Stepglm, or Stepwise Generalized Linear Model, refines model accuracy by iteratively adding or removing predictors based on their statistical significance, optimizing the model for maximum performance (**Figure 4G**). These models demonstrated robust predictive power, achieving AUC values between 0.894 and 0.925 in the training set (**Figure 4G**). Notably, this high performance extended to the validation set, where all selected models achieved AUC values exceeding 0.8, with an average AUC of over 0.84 across both sets (**Figure 4G**). The strong accuracy of these models underscores the utility of advanced computational techniques in improving our understanding and management of T2DM, enabling precise subtype classification based on the metabolic profiles of T-cells.

### Drug enrichment analyses for personalized treatment

To facilitate the application of the three subtypes of T2DM for personalized treatment, a drug enrichment analysis was conducted on the upregulated DEGs (logFC > 0.5) for each subtype. This approach identifies potential drugs tailored to the specific needs of each T2DM subtype, offering a foundation for more targeted therapeutic strategies.

### Subtype A: suloctidil and inflammation pathways

**Figure 5:**
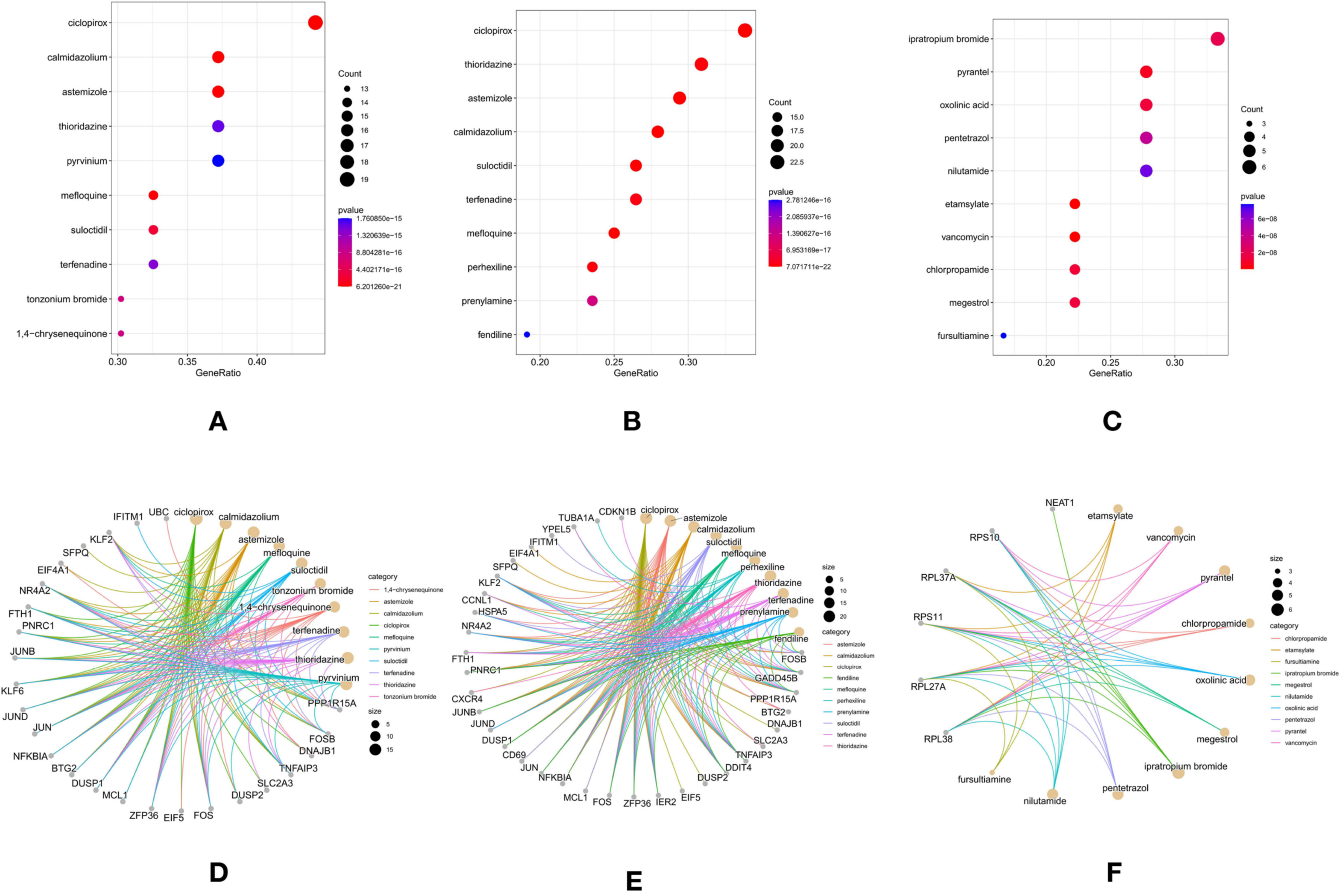
Drug Enrichment Analyses in T2DM Subtypes: **(A)** Dot plot showing the top 10 lowest P-value enriched drugs in Subtype A. **(B)** Dot plot showing the top 10 lowest P-value enriched drugs in Subtype B. **(C)** Dot plot showing the top 10 lowest P-value enriched drugs in Subtype C. **(D)** Cnetplot showing the correlated genes with enriched drugs in Subtype A. **(E)** Cnetplot showing the correlated genes with enriched drugs in Subtype B. **(F)** Cnetplot showing the correlated genes with enriched drugs in Subtype C.

In subtype A, suloctidil emerged as the most promising drug for diabetes treatment (**Figure 5A**) (23). This drug was linked to genes involved in inflammation and immune regulation, including NR4A2, IFITM1, PPP1R15A, FOSB, TNFAIP3, FOS, ZFP36, MCL1, DUSP1, NFKBIA, JUN, KLF6, KLF2, and FTH1 (**Figure 5D**). These genes are critical in regulating inflammatory responses, which are central to insulin resistance and diabetes-related complications. The enrichment of suloctidil with these genes suggests its potential in managing the inflammation-associated aspects of T2DM in subtype A.

### Subtype B: suloctidil as a key drug

For subtype B, suloctidil was again identified as the key drug associated with diabetes treatment (**Figure 5B**) (23). This drug was linked to genes such as NR4A2, FOSB, GADD45B, IFITM1, PPP1R15A, TNFAIP3, DDIT4, IER2, ZFP36, FOS, MCL1, NFKBIA, HSPA5, JUN, CD69, DUSP1, KLF2, and FTH1 (**Figure 5E**), which are involved in stress responses, immune regulation, and cell survival. While suloctidil remained the most relevant drug for this subtype, other medications, such as Fendiline, Prenylamine, and Perhexiline—though primarily used for cardiovascular issues—may have indirect effects on diabetes, but are not specifically designed for its treatment.

### Subtype C: chlorpropamide for insulin regulation

In subtype C, chlorpropamide was identified as the key drug associated with diabetes treatment (**Figure 5C**). As a sulfonylurea, chlorpropamide stimulates insulin secretion, which plays a pivotal role in improving glucose control in patients with T2DM. This drug was associated with genes such as GADD45B, PPP1R15A, TNFAIP3, DDIT4, IER2, ZFP36, FOS, HSPA5, JUN, DUSP1, and KLF2 (**Figure 5F**), which are involved in stress response and metabolic regulation. These associations suggest that chlorpropamide may be particularly effective in managing insulin secretion and glucose metabolism in subtype C.

## Discussion

T2DM is a complex metabolic disorder marked by chronic hyperglycemia resulting from insulin resistance and impaired insulin secretion (24). This study sought to investigate the immunological and metabolic alterations in T2DM by analyzing single-cell RNA sequencing data from PBMCs of patients with T2DM and HC. Our findings highlighted significant immune cell alterations, including an increase in monocytes and a decrease in CD4+ T cells in patients with T2DM. Furthermore, we observed metabolic heterogeneity within T cell subpopulations and enhanced cell-cell communication pathways in T2DM.

The observed increase in monocytes in patients with T2DM reflects heightened chronic inflammation and immune activation (4). These monocytes contribute to insulin resistance by secreting pro-inflammatory cytokines such as TNF-α and IL-6 (8). Previous studies have shown that monocyte-derived macrophages infiltrate adipose tissue in T2DM, where they play a pivotal role in promoting inflammation and exacerbating insulin resistance (25, 26). In contrast, the decrease in CD4+ T cells, which are critical for coordinating adaptive immune responses, may impair immune regulation (27). This reduction in CD4+ T cells potentially undermines the body’s ability to control inflammation, exacerbating insulin resistance and beta-cell dysfunction (28).

Further alterations in T cell subtypes underscore the immune dysregulation associated with T2DM. An increase in cytotoxic CD8+ T cells and naive CD8+ T cells likely reflects an overactive immune surveillance mechanism (29). Elevated cytotoxic CD8+ T cells can induce beta-cell apoptosis, impairing insulin secretion (30). The rise in naive CD8+ T cells indicates ongoing recruitment and activation in response to chronic metabolic stress (31). These changes suggest an altered immune response, compromising the body’s ability to regulate inflammation and immune tolerance, thereby contributing to the pathogenesis of T2DM (32, 33). The reduction in CD4+ T cells, which are critical for orchestrating adaptive immune responses, may compromise immune regulation. Previous studies have linked decreased CD4+ T cell counts in patients with T2DM to impaired immune tolerance and increased autoimmunity (34, 35). This decline may result in unregulated inflammatory responses, thereby exacerbating the chronic low-grade inflammation characteristic of T2DM (36).

Analysis of metabolic heterogeneity within T cell subpopulations revealed distinct metabolic profiles in patients with T2DM. Subtype A T cells exhibited high expression of lipid and amino acid metabolism pathways, suggesting an adaptive metabolic response to the diabetic environment (37). This subtype demonstrated broad metabolic activity, particularly in lipid, amino acid, and carbohydrate metabolism, indicating an adaptive response to chronic stress. However, it may also reflect a hyperactivated or exhausted T cell state. Such metabolic reprogramming likely enables T cells to survive in conditions of altered nutrient availability, but it could also promote a pro-inflammatory phenotype (38). Subtype B, with its emphasis on oxidative stress-related pathways, indicates a heightened immune response, while Subtype C displays more targeted metabolic reprogramming, suggesting a potentially less generalized immune activation (39). These metabolic alterations may affect T cell activation and function, potentially exacerbating immune dysfunction in T2DM (40). In contrast, the HC group exhibited baseline metabolic activity, emphasizing the metabolic disturbances present in patients with T2DM. Metabolic reprogramming significantly impacts T cell function (41), and understanding these shifts is critical for developing targeted therapies aimed at restoring normal T cell function and improving metabolic control.

Enhanced cell-cell communication pathways were also observed in patients with T2DM, indicating intensified immune responses. CellChat analysis revealed heightened activity of pathways such as CD30, CD48, TGF-β, and IFN-γ in subtype B (42). These pathways are pivotal in immune regulation, T cell activation, and cytokine signaling. CD30 activation, for instance, can drive pro-inflammatory responses and immune dysregulation, while TGF-β plays a key role in balancing immune tolerance and inflammation. In the context of T2DM, the activation of these pathways may impair immune function, exacerbate insulin resistance, and contribute to beta-cell dysfunction (43). Furthermore, alterations in TNF and CCL pathway engagement, critical for inflammation and immune cell recruitment, suggest significant changes in chemokine signaling, which could further influence immune cell interactions and disease progression (44).

The dominance of the MHC-I pathway emphasizes the importance of antigen presentation in T2DM (45). Increased antigen presentation may enhance autoimmune responses, potentially contributing to beta-cell destruction (46). The selective activation of the visfatin pathway in specific T2DM subtypes may reflect unique metabolic and inflammatory states, providing potential targets for subtype-specific interventions (47). Focusing on the MHC-I and visfatin pathways could offer targeted therapeutic opportunities for more effective management of T2DM. Collectively, these findings underscore the complexity of immune cell interactions in T2DM and highlight potential pathways for therapeutic targeting.

This study also highlighted the critical role of TFs in regulating immune cell function and metabolic processes in T2DM. We identified several TFs that are differentially expressed across T2DM subtypes, including those involved in immune response regulation and insulin resistance. Notably, TFs such as NF-kB and STAT3, key players in inflammatory pathways, were upregulated in patients with T2DM, highlighting the persistent immune activation and inflammatory environment characteristic of the disease (48). In contrast, TFs associated with insulin signaling, such as PAX6 and FOXO1, were downregulated, potentially contributing to impaired insulin secretion and resistance (49, 50). The dysregulation of TF activity in T2DM thus opens novel therapeutic avenues, as targeting specific TFs could help restore immune homeostasis and improve metabolic control, offering a more tailored approach to treatment.

The drug enrichment analysis further reinforces the potential for personalized T2DM therapy based on TF activity and metabolic alterations. For example, suloctidil, identified as associated with specific immune-related TFs and inflammatory pathways, could serve as a promising candidate for managing inflammation and immune dysfunction in T2DM (8). Similarly, targeting pathways regulated by TFs like NF-kB and STAT3 may help reverse the chronic inflammation that drives T2DM pathogenesis (51, 52). Drugs such as chlorpropamide, which influence insulin secretion, may be especially effective for subtypes with dysregulated insulin signaling pathways (53). These findings emphasize the importance of integrating TF activity and drug enrichment data into personalized treatment strategies, potentially improving therapeutic outcomes by addressing the underlying molecular mechanisms specific to each patient’s disease profile.

Clinically, the development of advanced machine learning models enabled accurate classification of T2DM subtypes based on T cell metabolic profiles. These models achieved high AUC values, demonstrating their potential application in clinical settings for patient stratification and personalized treatment planning. By identifying distinct metabolic and immunological signatures linked to different T2DM subtypes, clinicians can better tailor interventions to address the underlying dysfunctions of each patient.

### Limitation

Despite the strengths of this study, several limitations remain. These include potential biases arising from the use of public datasets, a limited sample size that may impact generalizability, and the cross-sectional design, which limits causal inference regarding immune changes and T2DM progression. Additionally, findings may not be universally applicable due to demographic variations in T2DM influences. Inherent limitations of single-cell sequencing, such as dropout events and batch effects, may also impact data interpretation. Future research should validate these results using larger, more diverse cohorts, incorporate longitudinal studies to explore disease progression, and evaluate targeted therapies through clinical trials, with predictive models supporting personalized treatment strategies.

## Conclusion

In conclusion, this study highlights significant immune and metabolic dysregulation in T2DM, marked by elevated monocytes, reduced CD4+ T cells, and distinct metabolic profiles within T cell subpopulations. Enhanced cell-cell communication pathways, particularly those involving the MHC-I pathway, further highlight the complexity of the immune landscape in T2DM. The analysis of TF activity, in conjunction with drug enrichment findings, identifies promising therapeutic targets for personalized treatment. Integrating these immunological and metabolic insights—along with key TFs and drug candidates—into clinical practice could optimize T2DM management and improve patient outcomes, reinforcing the critical role of personalized medicine in addressing the multifaceted nature of metabolic disorders.

## Data Availability

The original contributions presented in the study are included in the article/**Supplementary Material**. Further inquiries can be directed to the corresponding author.

## Supplementary material

Supplementary Table 1 Cell-cell interactions between T cells and monocytes in Subtype A.

Supplementary Table 2 Cell-cell interactions between T cells and monocytes in Subtype B.

Supplementary Table 3 Cell-cell interactions between T cells and monocytes in Subtype C.

Supplementary Table 4 Cell-cell interactions between T cells and monocytes in Healthy Controls.
